# Single-Walled (Magnetic) Carbon Nanotubes in a Pectin Matrix in the Design of an Allantoin Delivery System

**DOI:** 10.1021/acsomega.3c03619

**Published:** 2024-02-21

**Authors:** Ö. Zeynep Güner Yılmaz, Anıl Yılmaz, Serdar Bozoglu, Nilgun Karatepe, Saime Batirel, Ali Sahin, Fatma Seniha Güner

**Affiliations:** † Department of Chemical Engineering, Istanbul Technical University, Maslak, Istanbul 34469, Turkey; ‡ Energy Institute, Renewable Energy Division, Istanbul Technical University, Maslak, Istanbul 34469, Turkey; § Department of Biochemistry, Faculty of Medicine, Marmara University, Istanbul 34854, Turkey; ∥ Genetic and Metabolic Diseases Research Center (GEMHAM), Marmara University, Istanbul 34854, Turkey; ⊥ Sabancı University Nanotechnology Research and Application Center (SUNUM), Sabancı University, Istanbul 34956, Turkey

## Abstract

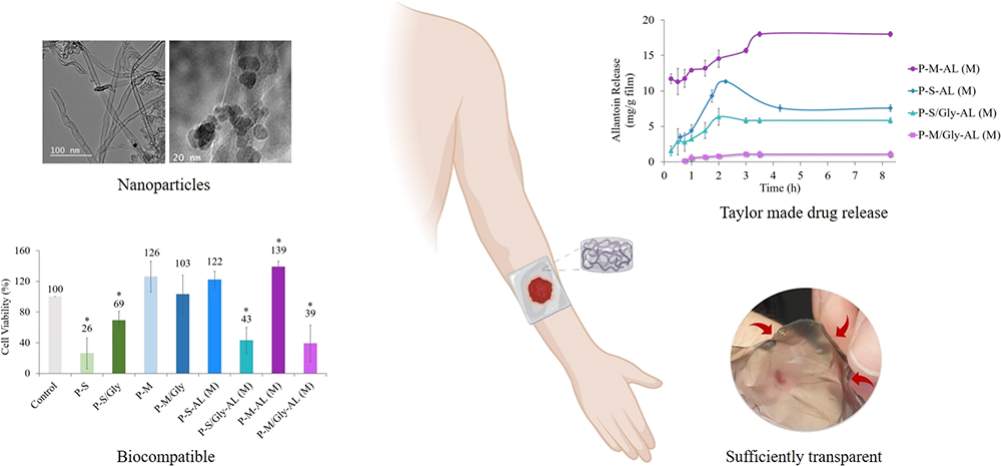

Single-walled carbon nanotubes (SWCNTs) outperform other materials due to their high conductivity, large specific surface area, and chemical resistance. They have numerous biomedical applications, including the magnetization of the SWCNT (mSWCNT). The drug loading and release properties of see-through pectin hydrogels doped with SWCNTs and mSWCNTs were evaluated in this study. The active molecule in the hydrogel structure is allantoin, and calcium chloride serves as a cross-linker. In addition to mixing, absorption, and swelling techniques, drug loading into carbon nanotubes was also been studied. To characterize the films, differential scanning calorimetry (DSC), thermal gravimetric analysis (TGA), Fourier transform infrared (FTIR) spectroscopy, surface contact angle measurements, and opacity analysis were carried out. Apart from these, a rheological analysis was also carried out to examine the flow properties of the hydrogels. The study was also expanded to include *N*-(9-fluorenyl methoxycarbonyl)glycine-coated SWCNTs and mSWCNTs as additives to evaluate the efficiency of the drug-loading approach. Although the CNT additive was used at a 1:1000 weight ratio, it had a significant impact on the hydrogel properties. This effect, which was first observed in the thermal properties, was confirmed in rheological analyses by increasing solution viscosity. Additionally, rheological analysis and drug release profiles show that the type of additive causes a change in the matrix structure. According to TGA findings, even though SWCNTs and mSWCNTs were not coated more than 5%, the coating had a significant effect on drug release control. In addition to all findings, cell viability tests revealed that hydrogels with various additives could be used for visual wound monitoring, hyperthermia treatment, and allantoin release in wound treatment applications.

## 1 Introduction

Carbon nanotubes (CNTs) are one of the nanoparticle groups used in biomedical applications^1−5^ and the food industry.^6^ Even though their toxic effect is a matter of debate,^7,8^ interest in carbon-based nanomaterials continues to increase. Among carbon-based nanomaterials, single-walled CNTs (SWCNTs) have been widely used in drug delivery systems for the last decades^9−17^ due to their low cytotoxicity compared to the other type of CNTs.^8^ Thanks to their large surface area, unique structure, and excellent physical properties, they are among the best nanomaterials for highly effective drug and biomolecule delivery. They can be conjugated noncovalently or covalently with drugs, biomolecules, and nanoparticles.^18−20^ Also, due to their extremely wide surface area, a variety of molecules can multiconjugate on their sidewalls. Aromatic-group molecules can be easily noncovalently bound to CNTs via strong π–π interactions.^19,20^ 1D functionalized CNTs (f-CNTs) could enhance the binding to a molecule by interacting through several binding sites due to their flexibility.^21^

Magnetic carbon nanotubes (mCNTs) have been developed to impart magnetic properties to CNTs. Because of the high enrichment performance and magnetic properties,^22,23^ mCNTs are widely used not only in biomedical applications such as drug delivery agents,^24−26^ and magnetic resonance imaging^27^ but also in chemical and environmental purposes as magnetic solid-phase extraction adsorbents,^28,29^ removal of heavy metals and dyes from water,^30−33^ water treatment,^34,35^ sensors,^36,37^ and catalysis.^38^ Compared with other nanoparticles, magnetically aided drug delivery and hyperthermia treatment are the most important contributions of magnetic nanoparticles for the diagnosis and therapy of damaged tissues.^39−42^

Despite their superior features, CNTs have some disadvantages. Due to their low solubility in aqueous media, they tend to collapse because pristine CNTs have hydrophobic structures. This is a serious problem, especially for biomedical applications. There are a few strategies that are useful for solving solubility problems, such as the functionalization of the CNT surface with hydrophilic macromers/polymers by covalent bonds^43^ or noncovalent interactions.^19,20,44,45^ Noncovalent methods are preferable to obtain functionalized CNTs in various applications since the structure of carbon nanotubes is impaired after the chemical reaction in covalent modification, so their inherent physical properties are destroyed.^46,47^

On the other hand, pectin has great potential in multipurpose applications in the food and healthcare industries due to its biocompatibility, antimicrobial, and anti-inflammatory properties.^48^ Prebiotic, hypoglycemic, hypocholesterolemia, and anticancer effects of pectin in the human body are reported by researchers in the literature.^48,49^ By cross-linking in the presence of a divalent cation, low methoxy pectin forms a porous hydrogel structure. Due to its superior properties, pectin hydrogels can be used in biomedical applications including tissue engineering, drug delivery systems applied via the nasal, oral, or ocular routes, cancer-targeted drug delivery, gene delivery, and wound healing.^50−52^ Loading active molecules into the pores, which takes advantage of the high swelling property, enables the development of drug-release systems. The major disadvantage in the design of a pectin hydrogel system is the low mechanical strength of pectin, so it needs improvement.^53^ One of the strategies used to enhance its mechanical properties is the addition of nanoparticles or small organic molecules to the pectin matrix. In this way, while the mechanical properties of the pectin matrix become resistant against forces, drug release behaviors,^54−57^ antimicrobial activity,^58^ and bioactive ability^59^ also improved, i.e., two birds with one stone.

The individual advantages of pectin and CNTs/mCNTs as drug delivery systems encouraged us to prepare a new drug-containing wound dressing from pectin and the SWCNT or magnetic SWCNT (mSWCNT) and to explore their synergistic effects on the system. Allantoin, which promotes wound healing, was chosen as the model drug. With the dispersion of SWCNTs or mSWCNTs in the pectin matrix, we aimed to improve the mechanical integrity and drug release performance of the system. Additionally, the introduction of mSWCNTs into the system allows for the observation of the influence of iron oxide particles attached to the SWCNTs on the enhancement of the drug release capabilities of the hydrogel. Moreover, in order to mitigate the potential toxic effects of carbon nanotubes dispersed within the pectin matrix, a noncovalent coating strategy was employed using a fluorenyl methoxycarbonyl (Fmoc) functionalized amino acid to modify the CNT walls. According to the data obtained, the drug release behavior of the system could be improved by Fmoc-coated CNTs, but the cytotoxicity is not. On the other hand, very promising results were obtained for the cytotoxicity of samples prepared with the uncoated-CNTs.

## 2 Materials and Methods

Amidated low-methoxy pectin (degree of esterification = 31%) was provided by Herbstrith & Fox company (Neuenbürg, Germany). Glycerol (purity-99%) and CaCl_2_·2H_2_O were supplied by Labkim (Istanbul, Turkey). Allantoin (AL) was provided by Kalekim (Istanbul, Turkey). *N*-(9-Fluorenyl methoxycarbonyl) glycine (Fmoc-Gly-OH) was supplied by Sigma-Aldrich. Tetrahydrofuran (THF) was supplied by J.T. Baker. Double-deionized water was used throughout the experiments.

### 2.1 Synthesis of SWCNTs

Single-walled carbon nanotubes were synthesized using the fluidized-bed chemical vapor deposition (CVD)^60^ of acetylene (C_2_H_2_) on magnesium oxide (MgO) powder impregnated with an iron nitrate [Fe(NO_3_)_3_·9H_2_O] solution. Briefly, the CVD apparatus consisted of a vertical furnace and a quartz glass tube with a diameter of 3 cm in the middle of a quartz filter. A magnesium oxide (100 m^2^/g) supported iron oxide powder produced by impregnation in an iron nitrate ethanol solution was used as a precursor powder. Then, the furnace was heated to the synthesis temperature (800 °C) and thus iron oxide clusters were formed because of the thermal decomposition of the iron nitrate at 125 °C. The synthesis started with the introduction of acetylene mixed with argon and lasted for 30 min. After synthesis, SWNTs were purified with 6 M HNO_3_ for 3 h.

### 2.2 Synthesis of mSWCNTs

**Figure 1:**
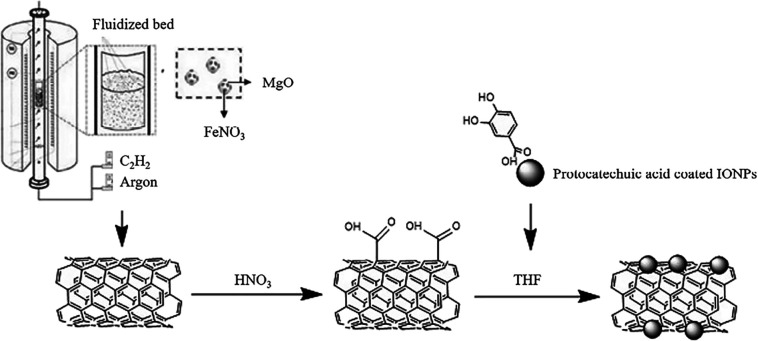
General scheme of SWCNT production and functionalization.

Iron oxide nanoparticles (IONPs) were synthesized and coated with protocatechuic acid (PCA) similar to our previous study.^61^ Afterward, mCNTs were prepared by a ligand exchange method. SWCNTs were dispersed for 30 min in 20 mL of THF with the help of an ultrasonic probe. A second solution was prepared in 15 mL of THF with PCA-coated IONPs. This solution was added to the SWCNT solution with a weight ratio of 2/1 (SWCNTs/IONPs) and stirred at 700 rpm for 6 h. The suspension was filtered by using a 1 μm pore size membrane. The nanotubes on the filter were dispersed in THF, sonicated in a water bath for 10 min, and then filtered. This sequence was repeated with deionized water to eliminate unbound and free PCA molecules. Finally, the nanotubes were separated from the liquid phase by using a magnet and freeze-dried for 24 h. A scheme of SWCNTs production and functionalization is seen in Figure 1.

### 2.3 Coating of CNTs with Fmoc-Gly-OH

50 mg of SWCNT or mSWCNT was dispersed in THF for 30 min using an ultrasonic bath. Then, 10^–3^ mol Fmoc-Gly-OH was weighed and put into the dispersed SWCNTs or mSWCNTs. After 7 days, the solution was filtered by using a polytetrafluoroethylene (PTFE) filter with a pore size of 0.2 μm. The obtained powder was dried for 24 h.

### 2.4 Preparation of Drug-Loaded Hydrogels

To determine the effect of the preparation method on the drug release performances of the samples in preliminary studies, CNT-added hydrogels were prepared in four different methods; mixing, swelling, absorption, and direct methods. For this purpose, SWCNTs were used. After the determination of the preparation method, coated or uncoated mSWCNTs were added to the pectin matrix using the selected method. Other hydrogels were also synthesized as comparison samples at various stages of the study. The codes and components of the hydrogels synthesized in this study are given in Table 1.

**Table 1:** Codes and Components of the Hydrogels

Code	Nanoparticle	Allantoin	Matrix	Method
P	–	–	Pectin	–
P–S	SWCNT	–	Pectin	–
P–S/Gly	Fmoc-Gly-OH coated SWCNT	–	Pectin	–
P-M	mSWCNT	–	Pectin	–
P-M/Gly	Fmoc-Gly-OH coated mSWCNT	–	Pectin	–
P–S-AL	SWCNT	+	Pectin	Direct
P–S-AL (A)	SWCNT	+	Pectin	Adsorption
P–S-AL (S)	SWCNT	+	Pectin	Swelling
P–S-AL (M)	SWCNT	+	Pectin	Mixing
P-M-AL (M)	mSWCNT	+	Pectin	Mixing
P–S/Gly-AL (M)	Fmoc-Gly-OH coated SWCNT	+	Pectin	Mixing
P-M/Gly-AL (M)	Fmoc-Gly-OH coated mSWCNT	+	Pectin	Mixing

#### 2.4.1 Direct Method

Using an ultrasonic bath, 1 mg of SWCNT was dispersed in 10 mL of allantoin solution (3 mg/mL) for 30 min. Then, the solution was shaken at 100 rpm on an orbital shaker. After 2 h, the mixture was filtered by a PTFE filter with a pore size of 0.2 μm. After drying for 24 h, the obtained drug-loaded nanotubes were dispersed in 10 mL of ultrapure water and added into 30 g of pectin solution (2% w/w). The mixture was mechanically stirred for 24 h. Glycerol solution (5 wt %/w) was added to the mixture and stirred for 2 h at 100 rpm. Finally, it was cross-linked using 10 mL of CaCl_2_ solution (0.7% w/w) and a film was formed by drying at 100 rpm and 28 °C for 24 h.

#### 2.4.2 Mixing Method

30 g portion of pectin solution (2% w/w) was mixed with glycerol solution (5% w/w) added to the mixture and stirred for 2 h at 100 rpm. 30 mg of allantoin was added as 10 mL of pectin solution in this step, and the mixture was left to stir for 24 h. Then, 1 mg of SWCNT was dispersed in ultrapure water for 30 min using an ultrasonic bath and added to the mixture to stir for 24 h. After a homogeneous mixture was prepared, it was cross-linked using 10 mL of CaCl_2_ solution (0.7% w/w) and thus a film was formed as a result of drying at 100 rpm and 28 °C for 24 h.

#### 2.4.3 Swelling Method

30 g of pectin solution (2% w/w) was mixed with glycerol solution (5% w/w) added to the mixture and stirred for 2 h at 100 rpm. Then, 1 mg of SWCNT was dispersed in ultrapure water for 30 min using an ultrasonic bath and added to the mixture to stir for 24 h. After a homogeneous mixture was prepared, it was cross-linked using 10 mL of CaCl_2_ solution (0.7% w/w), and thus a film was formed as a result of drying at 100 rpm and 28 °C for 24 h. 10 mL of allantoin solution was added to the dry film in a Petri dish. In this way, after drying for 24 h, a drug-loaded film was obtained by the swelling method.

#### 2.4.4 Adsorption Method

30 g of pectin solution (2% w/w) was mixed with glycerol solution (5% w/w) added to the mixture and stirred for 2 h at 100 rpm. Then, 1 mg of SWCNT was dispersed in ultrapure water for 30 min using an ultrasonic bath and added to the mixture to stir for 24 h. After a homogeneous mixture was prepared, it was cross-linked using 10 mL of CaCl_2_ solution (0.7% w/w). 6 h after cross-linking, while the film was not yet completely dry, 10 mL of allantoin solution was added and the film continues to dry at 28 °C and 100 rpm.

### 2.5 Drug Release

The drug release study was accomplished with hydrogel films immersed in a 10 mL of buffer solution with a pH of 6.4. The films, weighing 18 ± 2 mg, were maintained at 26 ± 1 °C with agitation at 100 rpm. Following immersion of the films in the buffer, the amount of allantoin released from the films into the solution was determined up to 8.5 h by measuring the absorbance using UV spectroscopy at 210 nm.

### 2.6 Basic Characterization

Fourier-transform infrared (FTIR) spectroscopy was carried out on a Spectrum One model FTIR spectrometer (PerkinElmer, Connecticut, USA) between 650 and 4000 cm^–1^ using the attenuated total reflection (ATR) mode. DXR Raman model of Thermo Scientific with 532 nm laser was used in the study. Raman analysis is carried out at room temperature without preparation. TEM images of samples were taken on JEOL/JEM ARM 200 with an acceleration voltage of 200.0 kV. Thermal gravimetric analyses (TGA) were carried out under a nitrogen atmosphere using a Diamond model TGA (PerkinElmer, Massachusetts, USA) in the temperature range of room temperature to 800 °C with a 10 °C/min heating range under nitrogen. A PerkinElmer 4000 differential scanning calorimetry (DSC, PerkinElmer, Massachusetts, USA) was used to determine the thermal properties of the samples under a nitrogen atmosphere at −10 to 150 °C with a 10 °C/min screening rate. PerkinElmer UV–vis lambda 35 Spectrophotometer (PerkinElmer Corp, Waltham, MA, USA) was used to detect the opacity of the hydrogels. 10 mm wide samples were cut and placed in the chamber. %Transmittance was measured at 550 nm and the opacity was calculated by using eq 1.^6,62^
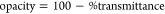
1

### 2.7 Rheological Analysis

Dynamic rheological measurements of swelled pectin hydrogels are performed using an Anton Paar (Graz, Austria) Physica Rheometer MCR301. To ensure compatibility with the 25 mm-wide and 1 mm-thick hydrogels, a 25 mm plate–plate geometry was employed for rheological measurements at room temperature. While the frequency is varied from 0.1 to 100 rad/s, the storage modulus (*G*′), the loss modulus (*G*″), and the damping factor (tan δ = *G*′′/*G*′) are determined. In addition, viscosity trends of pectin solutions with increasing shear rates from 1 to 100 Pa are determined using a 25 mm cone–plate geometry head.

### 2.8 Swelling

A hydrogel sample of 8 mm in diameter was cut and weighed. Then, they were placed in 50 mL of pH 6.4 Tris buffer and added to separate Petri plates about to be reweighed at determined intervals. The swelling of samples was calculated according to eq 2.
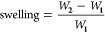
2where *W*_1_ is the dry polymer weight (g) and *W*_2_ is swelled polymer weight (g).

### 2.9 Cell Viability

The effect of hydrogels on cell viability was measured using the WST-1 assay. The assays were carried out with PCS-201–012 human dermal fibroblast cells, which were cultured in DMEM medium (PAN-Biotech, Germany) with 10% FBS (Biochrom, Germany) and 1% penicillin/streptomycin (PAN-Biotech, Germany) and maintained at 37 °C in air containing 5% CO_2_. After cells were seeded in a 96-well plate and incubated for 24 h, they were treated with the hydrogels for 24 h. Following the incubation, a WST-1 reagent (10 μL/100 μL) was added to each well. The absorbance of the sample was measured using a microplate reader (Enspire 2300, PerkinElmer, MA, USA) at 450 nm. The cell viabilities of treated cells are expressed as a percentage of the cell viabilities of nontreated cells. Duplicates of samples were used in each experiment, and three independent experiments were performed. Comparison of the groups was performed using one-way analysis of variance (ANOVA) followed by the Bonferroni post hoc test. The results are shown as mean ± standard deviation. *p* values of <0.05 were considered statistically significant. Live/dead fluorescent staining was also performed using a ThermoFisher kit. PCS-201–012 were incubated for a 24-h period. Following this incubation, the cells were subjected to a 1 h treatment in a light-protected environment, utilizing a solution containing 4 mM calcein-AM and 2 mM ethidium homodimer-I. Subsequently, microscopic images of the cells were captured. The intensity of green fluorescence, which indicates the presence of live cells, was quantified using ImageJ software. The results were expressed as a percentage change relative to the control group. To assess the statistical significance of the observed differences, we conducted an unpaired *t* test with a two-tailed analysis, where statistical significance was defined as a *p* value less than 0.05.

## 3 Results and Discussion

This study aimed to design a new drug carrier system with nontoxic and controlled drug release for biomedical applications. The components of the system were chosen as SWCNT or mSWCNT nanoparticles as the drug nanocarrier, Fmoc-Gly-OH as the coating molecule to the CNT walls, allantoin as the model drug, and pectin as the polymer matrix. The final product was intended to be in the form of a hydrogel. In the first part of the study, the effects of CNTs type (SWCNT or mSWCNT) and the contribution of Fmoc-Gly-OH molecules (coated or uncoated nanocarrier) on some basic properties of the hydrogel were investigated. For this purpose, allantoin-free hydrogels prepared with the addition of coated-/uncoated-SWCNT or mSWCNT nanocarriers were characterized by investigating their water contact angles, opacity, thermal behavior, and flow behavior. Thus, the positive and negative effects of the additives were determined.

In the second step, allantoin- and SWCNT-loaded hydrogels were prepared by four different methods, and their allantoin release and swelling behaviors were investigated to determine the allantoin loading method.

In the last part of the study, the contribution of Fmoc-Gly-OH coating on allantoin release and cytotoxicity of CNT-added hydrogels was investigated using rheological analysis, release kinetics, and cell viability tests.

### 3.1 Characterization of mSWCNTs

**Figure 2:**
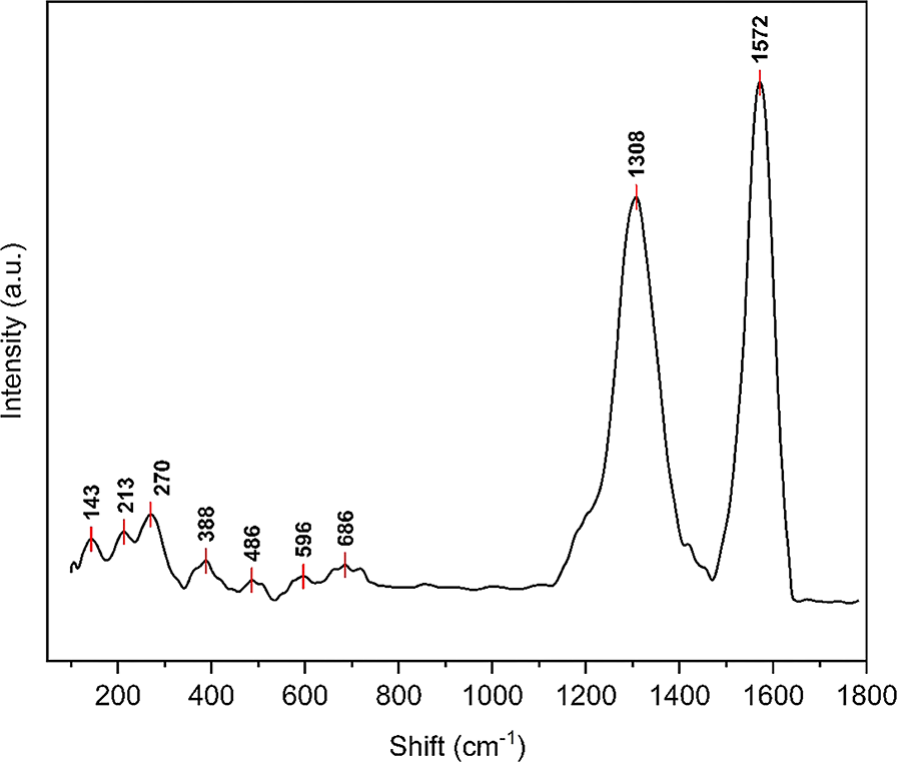
Raman spectra of mCNTs.

Chemical characterizations of CNTs were carried out using Raman and FTIR spectroscopy. Structural characterizations were performed using HR-TEM. The FTIR spectra of HNO_3_-treated SWCNTs can be seen in Figure S1. Raman spectra of the mCNTs can be seen in Figure 2. The peak at 685 cm^–1^, also known as the A1g mode, corresponds to the stretching vibrations of Fe^3+^ and O^2–^ in the tetrahedral site of the spinel structure in magnetite (Fe_3_O_4_). The group of peaks observed at approximately 213, 270, 387, 486, and 585 cm^–1^ belongs to the stretching vibrations of Fe and O in hematite (Fe_2_O_3_). The presence of Fe_2_O_3_ can be associated with the oxidation of magnetite to hematite owing to the high-power laser used during measurement.^63−65^ The peak at approximately 142 cm^–1^ is called the RBM mode and is the characteristic peak of the SWCNT structure.^66,67^

**Figure 3:**
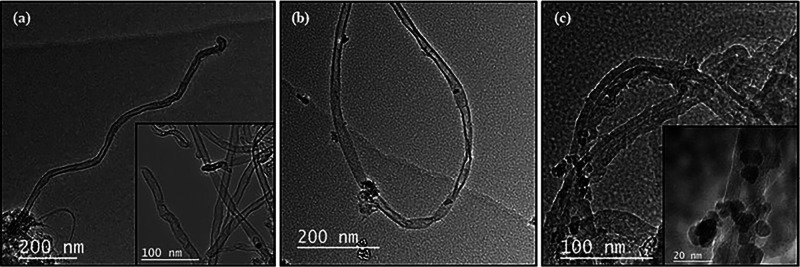
TEM images of (a) SWCNTs, (b) mSWCNTs, and (c) mSWCNT/Gly.

Figure 3a–c depicts the TEM images of SWCNTs, mSWCNTs, and mSWCNTs coated with Gly (mSWCNT/Gly), respectively. The formation of SWCNT is illustrated in Figure 3a, while Figure 3b,c shows iron oxide nanoparticles anchored onto the nanotube surface, distinguished by a darker color. These findings confirm the successful integration of iron oxide nanoparticles with SWCNTs, which is further corroborated by the presence of iron oxide peaks observed in the Raman spectra (Figure 2). TEM images of the iron oxide nanoparticles are shown in Figure S2. As shown in the images, the obtained nanoparticles had a spherical morphology. The size of the nanoparticles was measured from Figure S2a,b, and the average size was found as 9.94 nm. The crystallite size of the iron oxide nanoparticles was further calculated using the Scherrer equation and found to be 8.41 nm, which is consistent with the TEM results. The average width of carbon nanotubes and standard deviations were determined from Figure 3a,b and found as 15.32 nm and 4.08, respectively. It can be seen that the width of the carbon nanotubes is considerably larger than that of the values predicted for SWCNTs. We believe that this is because SWCNTs consist of large bundles and agglomerates owing to their large specific surface area and van der Waals attractions.^68^ The average length and standard deviation of carbon nanotubes were also determined as 1.06 and 0.124, respectively.

### 3.2 Allantoin-free Hydrogels

Four different hydrogels were successfully prepared by adding coated- or uncoated-SWNT or mSWCNT to the pectin matrix without allantoin, and their transparency, surface hydrophilicity, thermal behavior, and flow behaviors were investigated.

#### 3.2.1 Transparency and Surface Hydrophilicity of the Hydrogels

**Figure 4:**
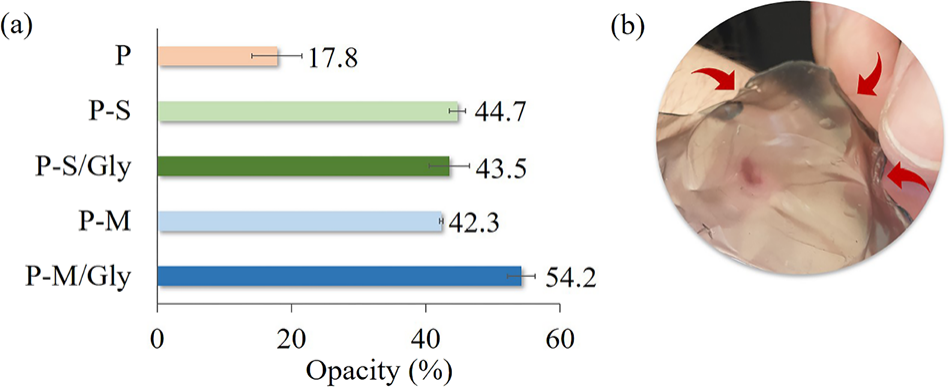
Opacity percent (a) and photograph (b) of the hydrogel.

When the opacity values of the prepared hydrogels were examined, it was determined that they increased from 16% to approximately 45% with the additive (Figure 4a). However, it can be seen that the hydrogel doped with SWCNTs is transparent (Figure 4b).

**Figure 5:**
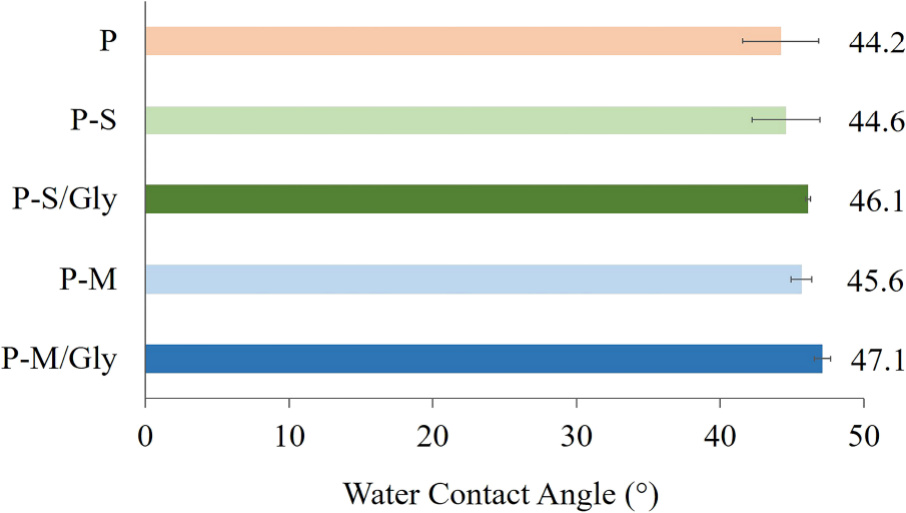
Water contact angle of the hydrogels.

The water contact angle is a basic characterization method for a material design for a biomedical application. The surface contact angles of the allantoin-free hydrogels were also determined in this study. According to the results, the water contact angles of all hydrogels are proximate to the hydrogel prepared without CNTs (Figure 5).

#### 3.2.2 Thermal and Flow Behaviors of the Hydrogels

**Figure 6:**
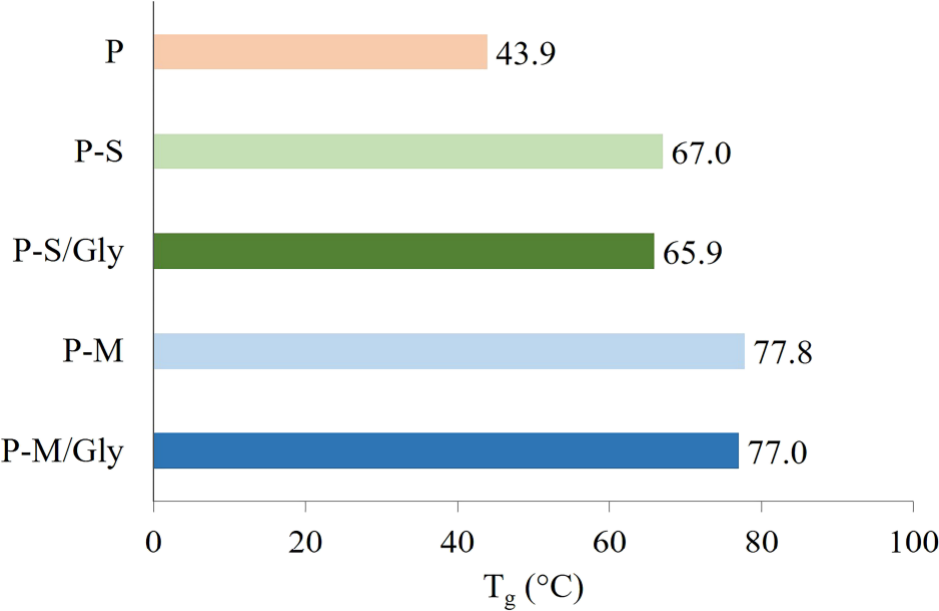
*T*_g_ values of the pectin hydrogels.

The glass transition temperature (*T*_g_) of the samples is shown in Figure 6. It is understood that the *T*_g_ value at which the polymer chains start to move increases with the addition of additives to the structure. This can be thought of as the additives restricting the chain mobility of the polymeric structure.^69^ Fmoc-Gly-OH coating is not an effective parameter for the *T*_g_ value. However, an increase of the *T*_g_ value is higher in the case of mSWCNT addition most likely due to the iron oxide content of the hydrogels. Our question here was why the mSWCNT nanoparticles increase the *T*_g_ of the hydrogel more than SWCNT nanoparticles. To provide additional data about thermal behavior, the thermogram data are presented in Figure S3.

**Figure 7:**
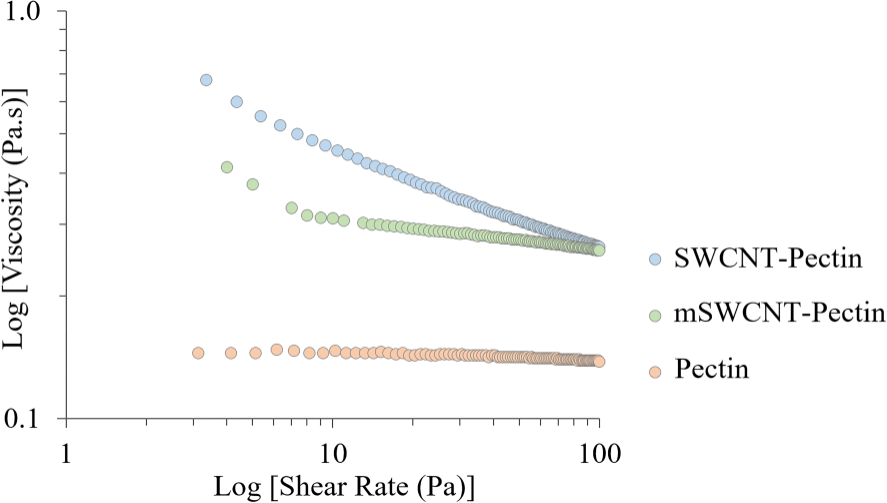
Viscosity of pectin, SWCNT-pectin, and mSWCNT-pectin solutions.

A rheological study was designed in an effort to find a new clue regarding this comment. A pectin solution, an SWCNT-added pectin solution, and an mSWCNT-added pectin solution were prepared, and their flow behaviors were investigated by a rotational rheometer. The amount of the nanoparticles was 1 mg in 30 mL of pectin solution. The viscosity curves of the three solutions are given in Figure 7. It is clearly seen that the viscosity increases when SWCNTs or mSWCNTs were added into the pectin solution due to the interactions between particle–particle and particle–polymer matrix, as reported in the literature.^70^ Moreover, one of the valuable data was obtained that the mSWCNT-pectin solution has the highest viscosity. According to the literature, both computational and experimental evidence shows that the carboxyl groups effectively form a covalent link with iron oxide in a pH 3–4 environment.^71−73^ In our case, a comparable interaction could potentially occur between the iron oxide groups within the magnetic carbon nanotube and the carboxylic groups present in the pectin. This interaction might take place during the 24 h mixing period, especially considering that the pH of the pectin solution was approximately 3.5 prior to the formation of the film. This interaction caused an increase viscosity. This finding also explains that the *T*_g_ value of the mSWCNT-added hydrogel is higher than that of the SWCNT-added hydrogel.

**Figure 8:**
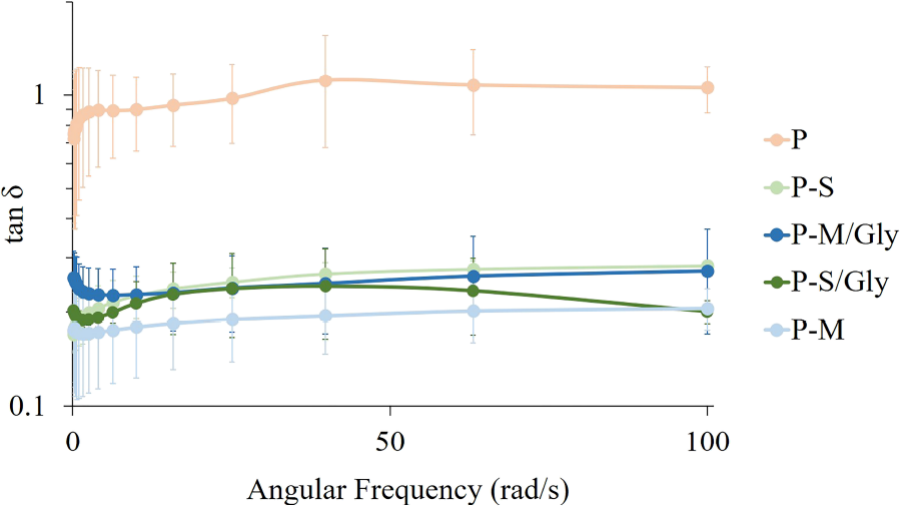
Tan δ of the swelled hydrogels.

Figure 8 shows the damping factor (tan δ = *G*″/*G*′) of each swelled hydrogel. *G*′ and *G*′′ denotes the storage and loss modulus, respectively. The tan δ of pectin hydrogel without CNT addition is significantly higher than the others. It is almost 1 or slightly higher than 1. On the other hand, CNT-added hydrogels prepared with mSWCNT addition have the lowest tan δ value. As reported in the literature,^74^ when the tan δ is higher than 1, liquid-like behavior is observed. On the contrary, when tan δ is lower than 1, a gel is in the solid state. Data obtained from rheological analyses were evaluated as CNT-added hydrogels exhibited solid-like behavior, unlike pectin hydrogel.

In conclusion, the type of CNT was an effective parameter for the thermal and flow behaviors of the hydrogels, but the Fmoc-Gly-OH coating did not affect it in any way.

### 3.3 Hydrogels Prepared from Allantoin-Loaded SWCNTs

Four different allantoin loading procedures were compared: mixing, swelling, adsorption, and direct methods. In this part of the study, the SWCNT was used as a nanocarrier. Allantoin was loaded into the samples at pH 4.6, and after 2 h, 100% efficient allantoin loading was achieved for all methods.

#### 3.3.1 Characterization

**Figure 9:**
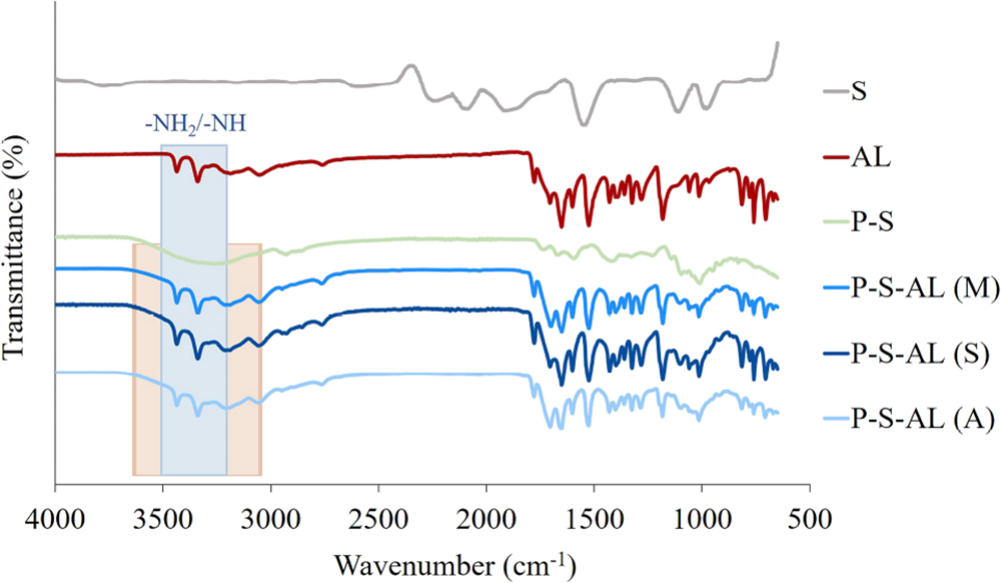
FTIR Spectra of SWNT (S), allantoin (AL), SWCNT-pectin films (P–S), and allantoin-loaded SWCNT-added hydrogels.

The presence of allantoin in the hydrogel matrix is demonstrated by FTIR spectroscopy. The amide groups of allantoin at 3500–3350 and 3450–3150 cm^–1^ related to the asymmetric and symmetric stretching vibrations^75^ and a wide −OH peak at about 3300 cm^–1^ from the pectin chains^76^ for allantoin-loaded hydrogels are seen in Figure 9. These data indicate that loading of allantoin into the pectin matrix was achieved.

#### 3.3.2 Swelling

**Figure 10:**
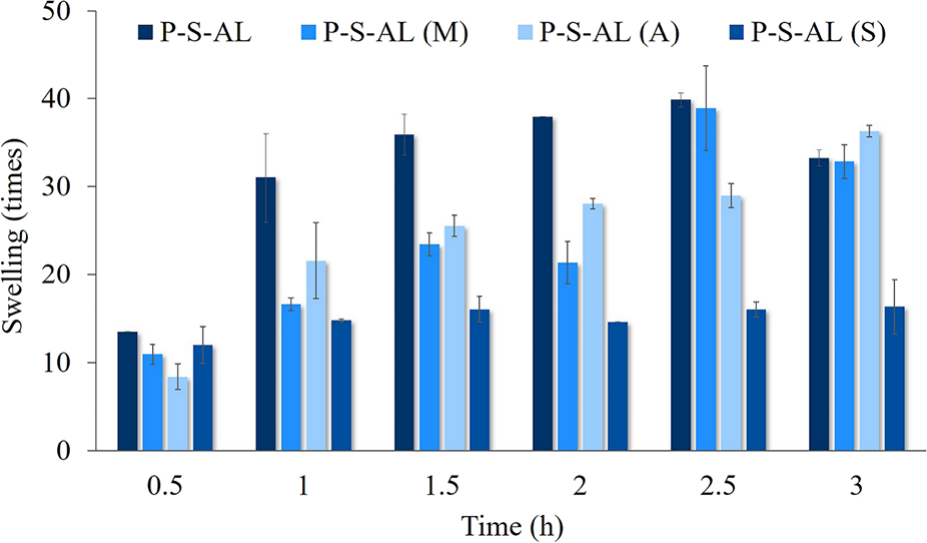
Swelling for allantoin- and SWCNT-added hydrogels.

The results of the swelling test for allantoin- and CNT-loaded hydrogels for up to three h are given in Figure 10. According to the obtained results, an increase was observed in the swelling values of the hydrogels prepared by mixing, adsorption, and direct method. In addition, the swelling values of the hydrogels prepared by the swelling method did not show a significant change. This result can be explained by the procedure followed during the preparation of the hydrogel.

In the swelling method, a hydrogel film was prepared from the pectin chain and calcium ions without adding allantoin. Here, the divalent calcium ion had the opportunity to engage in all possible interactions with the negative groups in the pectin structure during film preparation. Then, when the dry film was dipped in the allantoin solution, the film swelled and absorbed the allantoin. Meanwhile, the amine groups in the allantoin structure interacted with the exposed negative groups in the pectin structure, resulting in closer pectin chains. Therefore, when the hydrogel prepared with the swelling method was placed in Tris buffer solution for the swelling test, water molecules were less able to interact with the pectin chain due to the interaction of calcium ions and allantoin molecules with pectin chains.

#### 3.3.3 Allantoin Release

**Figure 11:**
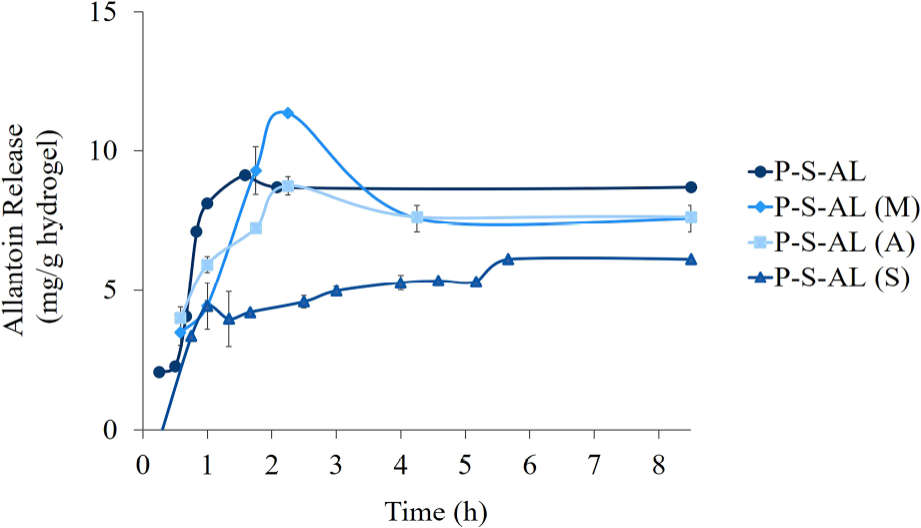
Allantoin release from hydrogels.

In the allantoin release studies conducted at pH 6.4, the amount of released allantoin within 8 h was found to be between 5.3 and 9.3 mg allantoin/g hydrogel. The amount of allantoin released did not change significantly according to the allantoin loading method, although there was a difference between the release profiles (Figure 11). Comparing the release profiles of all hydrogels, it is understood that the release becomes more controlled with the swelling method. Also, having similar release profiles, the hydrogels prepared by adsorption, direct, and mixing methods showed a burst release.

In the literature, a specific treatment dosage for wound healing has not been specified. On the other hand, the pharmaceutical formulations containing allantoin (Septalan, Alphosyl lotion) use a concentration of around 2%.^77^ Its positive effect on wound treatment has also been reported by many researchers. Araújo et al., for example, used a soft lotion O/W emulsion containing 5% allantoin for determination of its wound healing effect and they achieved promising results.^77^ A group of researchers prepared wound dressing films that release allantoin.^78^ Each film was loaded with approximately 3.4% allantoin by weight, and its in vitro kinetic release was investigated. They found 44% (1.5 mg) allantoin release in 12 h. Salas et al. also synthesized wound dressing films loaded with 1.2 mg allantoin/100 mg film.^79^ Their release performance was between 50 and 90 wt % (6–12 mg allantoin in release medium) in 120 h. In our research, we also obtained data on the same order of magnitude. In other studies, Ke et al. prepared a new nanoparticle for allantoin loading and release,^80^ and Yaşayan et al. encapsulated allantoin in composite films.^81^ They reported only a percentage of loading and release from nanoparticles, not a targeted allantoin amount.

To find the effect of the allantoin loading method on release profiles, we evaluated the results considering the swelling profiles of hydrogels. Concentration on the first 3 h of the release and swelling data, it is certainly seen that the swelling values tend to increase for 2.5 h like the release profile. Hence, we can conclude that the allantoin release occurs by the swelling of the hydrogel matrix as reported in the literature.^82^

The SWCNT-added hydrogel, prepared using the swelling method, can be utilized for controlled drug release in applications where precise control over the release is desired. However, in order to investigate the effect of Fmoc-Gly-OH in controlling the drug release, the mixing method with high burst release and convenience in terms of preparation method was chosen, and further studies were done.

### 3.4 Contribution of Fmoc-Gly-OH Coating on Allantoin Release

**Figure 12:**
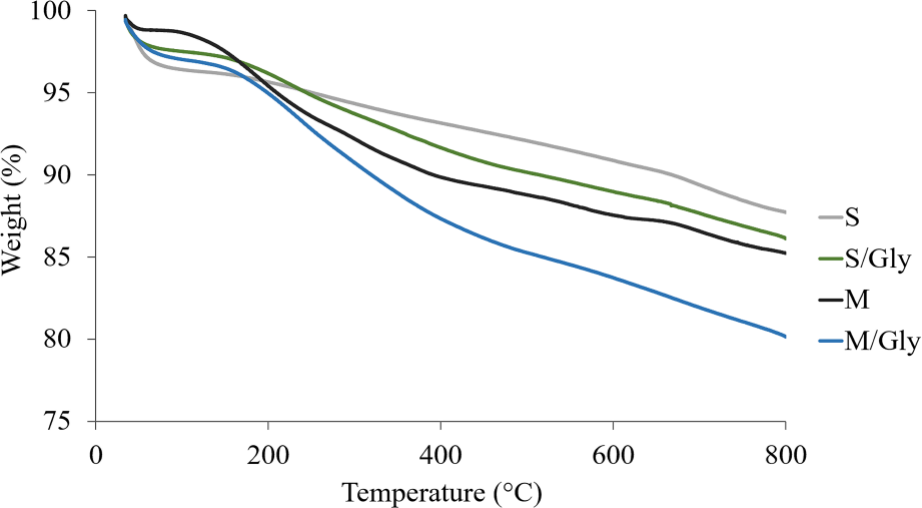
TGA curves of the coated and uncoated CNTs: SWNT (S), SWNT/Gly (S/Gly), mSWCNT (M), and mSWCNT/Gly (M/Gly).

To control the Allantoin release, nanotubes were coated with Fmoc-Gly-OH, and then they were dispersed in the pectin matrix by a mixing method. The amount of coating was determined by a TGA study. TGA curves of the coated- and uncoated-CNTs are seen in Figure 12. According to the obtained results, SWCNTs and mSWCNTs were coated with Fmoc-Gly-OH at 1% and 4.4%, respectively. However, an intriguing query arises as to why mSWCNTs exhibited a notably higher coating percentage compared to that of SWCNTs under identical conditions. At this point, it is conceivable that the elevated coating of mSWCNTs, approximately 3% more than that of SWCNTs, could be attributed to the presence of iron nanoparticles. This deduction resonates with the observation presented in the TEM image Figure 3c, where clusters of Fmoc-Gly-OH particles are noticeably aligned around magnetic nanoparticles. Within Figure 3c, specifically within the 20 nm-scaled view, the distinct cluster of Fmoc-Gly-OH particles surround the magnetic nanoparticle, visualized as light gray spheres. Simultaneously, on the exact opposite wall, there are distinct Fmoc-Gly-OH particles engaging directly with the nanotube surface.

#### 3.4.1 Allantoin Release

**Figure 13:**
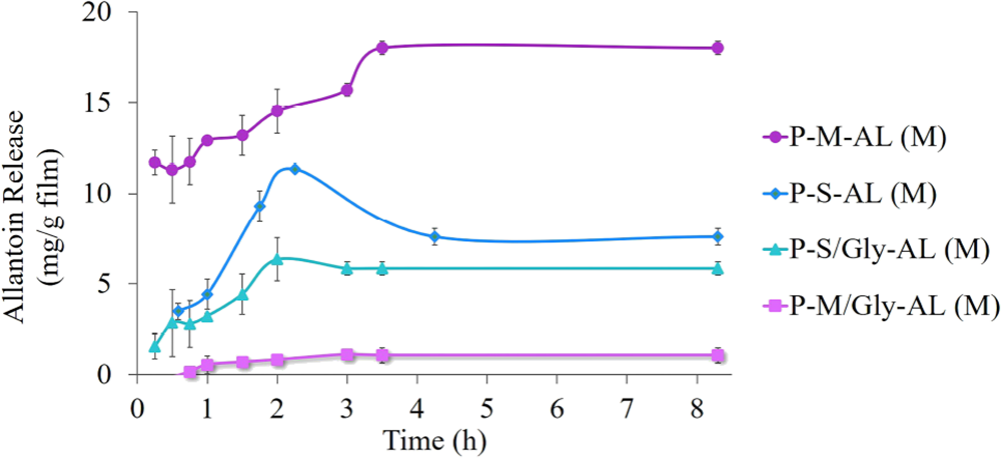
Allantoin release from hydrogels prepared by the mixing (M) method.

The effect of the nanotube type and coating with Fmoc-Gly-OH on allantoin release is shown in Figure 13. When the effect of the coating on the drug release profile is examined, we can clearly see the decrease in drug release of the coated nanotubes. As reported in the literature,^20^ CNT coating was achieved by noncovalent binding through π–π interaction between Fmoc groups and the hydrophobic CNT surface. Although the SWCNT was 1% coated with Fmoc-Gly-OH, a more controlled profile of drug release emerged than that of uncoated SWCNT. Interestingly, in the case of mSWCNT, even 4.4% Fmoc-Gly-OH coating caused an extremely controlled drug release. It is possible that the allantoin release pathway in the 3D hydrogel structure is extended by coating the CNT walls.

In the case of the uncoated nanoparticle-containing hydrogels, allantoin release is higher for the mSWCNT-added hydrogel than the SWCNT-added one. In the literature, it has been demonstrated through both theoretical calculations and experimental studies that Fmoc-protected amino acids can form complexes with iron through their carboxyl groups.^83^ Taking this into consideration along with the existing body of literature indicating the interaction between other carboxyl groups and iron oxide,^71−73^ it is conceivable that the carboxyl groups on pectin could also establish robust interactions with the magnetic particles on the nanotube surface. Therefore, this may have led to an increase in the distance between the polymer chains, resulting in a looser matrix. For SWCNT-added hydrogels, there is no possibility of an extra interaction. Thus, when compared with SWCNT addition, favorable conditions were created for uncoated-mSWCNT nanoparticles to create a less controlled release environment.

#### 3.4.2 Cell Viability Analysis

**Figure 14:**
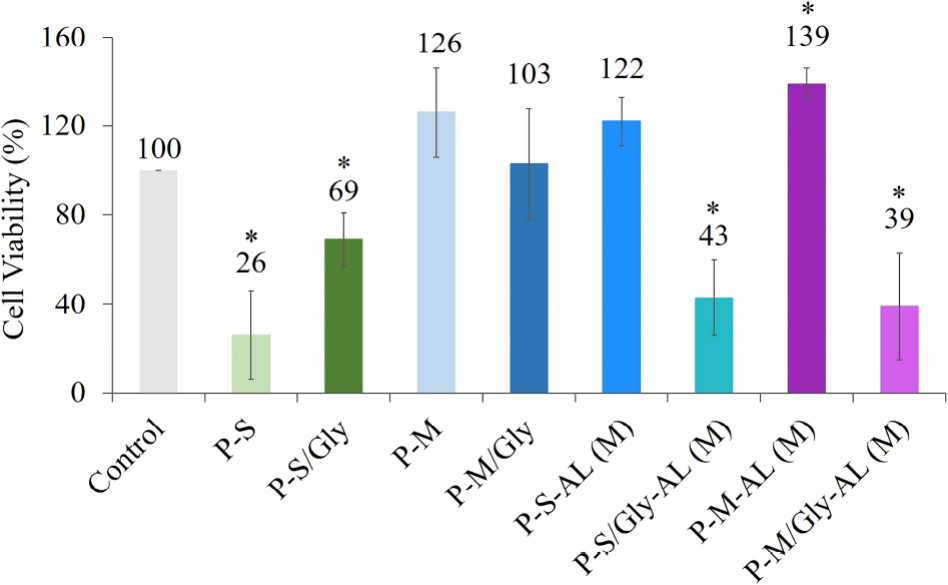
Cell viability analysis of the hydrogels.

Cell viability percentages of human dermal fibroblasts treated with hydrogels are shown in Figure 14. It was observed that the presence of SWCNTs in the pectin matrix decreased cell viability but this negative effect could be somewhat prevented by Fmoc-Gly-OH coating. However, mSWCNTs caused no harmful effects on cell viability due to the complex that might have been formed in the mSWCNTs between pectin and iron oxide nanoparticles. Therefore, except when drug release is necessary, mSWCNT-doped hydrogels can be used without adverse effects on cell viability. On the other hand, there was a variability in the effect of allantoin-loaded samples on cell viability. This variation might be due to the different secretion of allantoin by the hydrogels. There was a positive correlation between the amount of allantoin released and the percentage of viable cells.

Additionally, the viability of cells treated with allantoin-loaded hydrogels containing coated CNTs was lower than the viability of cells treated with allantoin-loaded hydrogels containing uncoated CNTs. It was assumed that noncovalently bonded Fmoc-Gly-OH molecules could leave the CNT walls to interact with the carboxyl groups of pectin in an acidic medium, CNT nanoparticles might have been unconfined and might have left the matrix, leading to an unexpected decrease in cell viability. Fmoc-Gly-OH was included in the CNT coating in our previous studies^20^ and did not reduce cell viability. This situation changes when added to the drug carrier pectin matrix because of the possible interaction between allantoin and Fmoc-Gly-OH.

Wu et. al argued that there exists an amide bond formation in the presence of methyl isonitrile at room temperature between carboxyl and amine groups of amino acids in a water medium at pH 3–8.^84^ As a result, since even a small percentage of Fmoc-Gly-OH coating is sufficient to control the release, coated CNTs can be used as biomaterials by increasing cell viability after an optimization study. In order to confirm the significant effect of P-M-AL (M) on cell viability, live/dead fluorescent staining was performed by using a ThermoFisher kit. Images of the cells were captured (Figure S4A-B). Parallel to the data of the WST-1 test, it was also observed that P-M-AL (M) significantly increased the cell viability to 128% (Figure S4C).

## 4 Conclusions

In this study, the main goal was to observe the effect of CNT derivatives on a pectin-originated hydrogel and to propose a new biomaterial for wound dressing applications. Throughout the study, we obtained transparent CNT-added stable pectin matrices. In addition, the coating of CNTs has been shown to significantly alter the drug release profile. P-M and P-M/Gly, which are nontoxic to cells and do not contain allantoin, can also be considered for visual wound monitoring and hyperthermia treatment. If drug-releasing dressings are desired to aid wound healing, then P-M-AL and P–S-AL may be preferred. Furthermore, if we must choose between them for a more controlled release of allantoin, then P–S-AL should be considered. After the interactions of Fmoc-Gly-OH in the matrix are thoroughly investigated, the biomaterial can be improved by conducting an optimization study to determine how much Fmoc-Gly-OH will be present in the system.

## Supporting Information Available

FTIR spectra of HNO_3_-treated SWCNTs (Figure S1); TEM images of iron oxide nanoparticles (Figure S2); thermograms of samples (Figure S3); fluorescence images from live/dead staining (Figure S4) (PDF)
