# Supplementary material for: Single-Walled (Magnetic) Carbon Nanotubes in a Pectin Matrix in the Design of an Allantoin Delivery System
Source: ACS Omega. 2024 Feb 21;9(9):10069–79. doi: 10.1021/acsomega.3c03619 (PMC10918663; doi:10.1021/acsomega.3c03619)
Supplement: Supplementary file 1 — ao3c03619_si_001.pdf [file ao3c03619_si_001.pdf]

Supporting Information

Single-walled (magnetic) CNTs in a pectin matrix in the design of an Allantoin delivery system

Ö. Zeynep Güner Yılmaz,[‡a] Anıl Yılmaz,[‡a] Serdar Bozoglu,[b] Nilgun Karatepe,[b] Ali Sahin,[cd] Saime Batirel[c] and Fatma Seniha Güner*[ae]

a. Department of Chemical Engineering, Istanbul Technical University, Maslak 34469 Istanbul, Turkey

b. Energy Institute, Renewable Energy Division, Istanbul Technical University, Maslak 34469 Istanbul, Turkey

c. Department of Biochemistry, Faculty of Medicine, Marmara University, 34854 Istanbul, Turkey

d. Genetic and Metabolic Diseases Research Center (GEMHAM), Marmara University, 34854 Istanbul, Turkey.

e. Sabancı University Nanotechnology Research and Application Center (SUNUM), Sabancı University, 34956 Istanbul, Turkey

‡ Authors contributed equally to this work.

* Corresponding author

Table of Contents

Figure S1. The FTIR spectra of HNO_3-treated SWCNTs.

Figure S2. TEM images of iron oxide nanoparticles a) 50 nm b) 20 nm.

Figure S3. Thermograms of the samples: P, P-S, P-S/Gly, P-M, and P-M/Gly.

Figure S4. Fluorescence images from live/dead staining. Fibroblast cells were cultured for 24 h with and without P-M-AL (M).

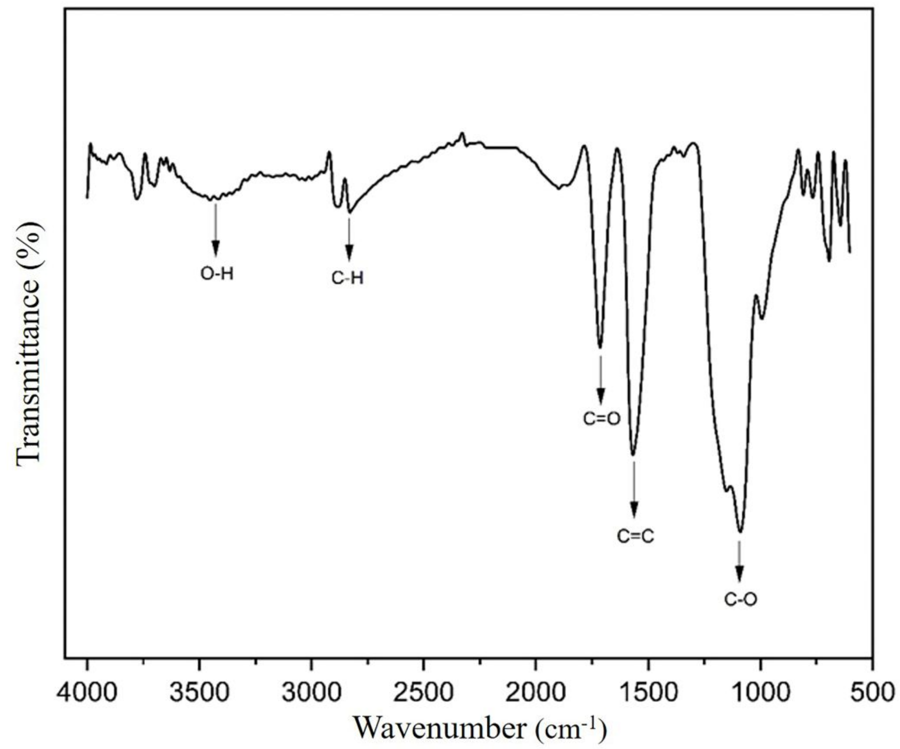

Figure S1. The FTIR spectra of HNO_3-treated SWCNTs.

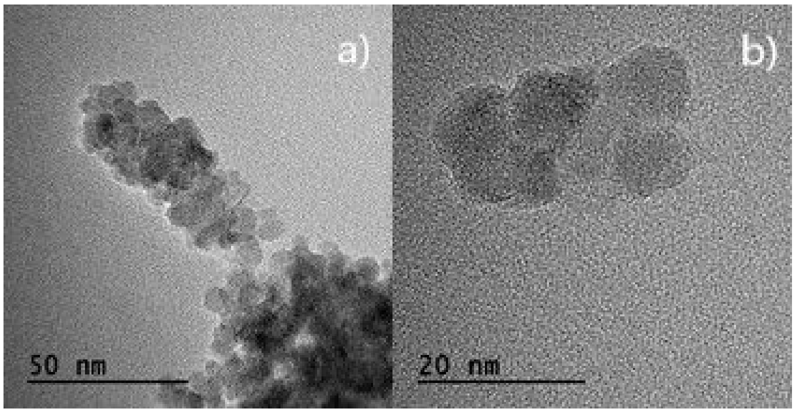

Figure S2. TEM images of iron oxide nanoparticles a) 50 nm b) 20 nm

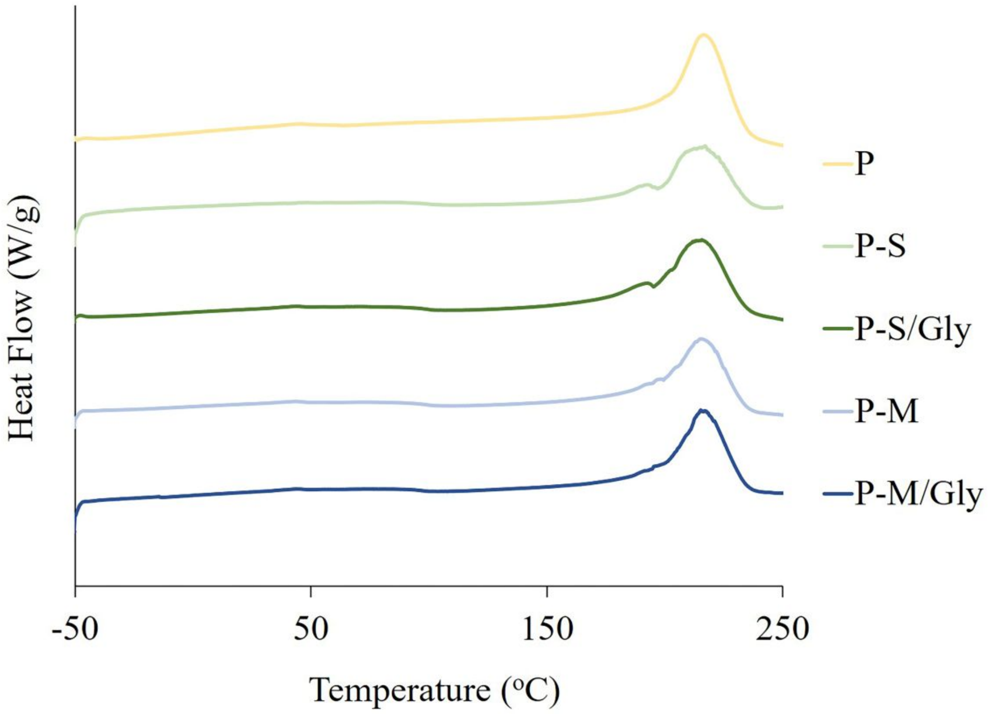

Figure S3. Thermograms of the samples: P, P-S, P-S/Gly, P-M, and P-M/Gly.

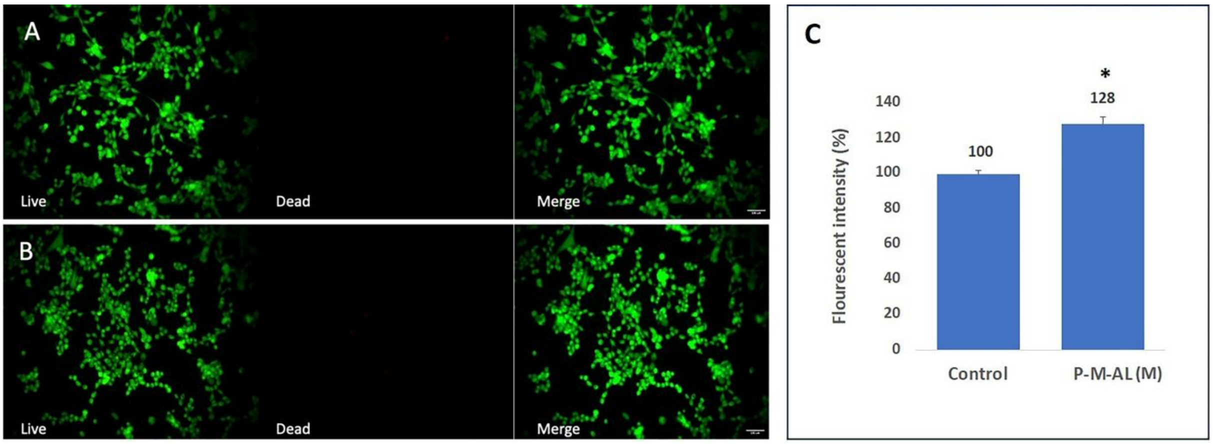

Figure S4. Fluorescence images from live/dead staining. Fibroblast cells were cultured for 24 h with and without P-M-AL (M). The merged images indicate live and dead cells together. Scale bar = 200 µm. **A)** Control **B)** P-M-AL (M) **C)** Green fluorescence intensity (Live cells) was quantified using Image J. Data shown are the mean ± SD, n = 5. * $P < 0.001$
